# Malaria in Venezuela: changes in the complexity of infection reflects the increment in transmission intensity

**DOI:** 10.1186/s12936-020-03247-z

**Published:** 2020-05-07

**Authors:** M. Andreína Pacheco, David A. Forero-Peña, Kristan A. Schneider, Melynar Chavero, Angel Gamardo, Luisamy Figuera, Esha R. Kadakia, María E. Grillet, Joseli Oliveira-Ferreira, Ananias A. Escalante

**Affiliations:** 1 Biology Department/Institute of Genomics and Evolutionary Medicine (iGEM), Temple University, Philadelphia, PA USA; 2 Escuela de Ciencias de la Salud, Universidad de Oriente, Núcleo Bolívar, Ciudad Bolívar, Venezuela; 3 Departamento de Medicina Interna, Complejo Hospitalario Universitario “Ruíz y Páez”, Ciudad Bolívar, Venezuela; 4 Biomedical Research and Therapeutic Vaccines Institute, Ciudad Bolívar, Venezuela; 5 Department CB, University of Applied Sciences Mittweida, Mittweida, Germany; 6 Instituto de Zoología y Ecología Tropical, Universidad Central de Venezuela, Caracas, Venezuela; 7 Institute Oswaldo Cruz, Oswaldo Cruz Foundation, Fiocruz, Rio de Janeiro, Brazil

**Keywords:** Drug resistance genes, *Pfdhps*, *Pfdhfr*, *Pfk13* gene, microsatellites, Multiplicity of infection, *Plasmodium falciparum*, *Plasmodium vivax*, Transmission intensity, Venezuela

## Abstract

**Background:**

Malaria incidence has reached staggering numbers in Venezuela. Commonly, Bolívar State accounted for approximately 70% of the country cases every year. Most cases cluster in the Sifontes municipality, a region characterized by an extractive economy, including gold mining. An increase in migration to Sifontes, driven by gold mining, fueled a malaria spillover to the rest of the country and the region. Here samples collected in 2018 were compared with a previous study of 2003/2004 to describe changes in the parasites population structures and the frequency of point mutations linked to anti-malarial drugs.

**Methods:**

A total of 88 *Plasmodium falciparum* and 94 *Plasmodium vivax* isolates were collected in 2018 and compared with samples from 2003/2004 (106 *P. falciparum* and 104 *P. vivax*). For *P. falciparum*, mutations linked to drug resistance (*Pfdhfr*, *Pfdhps*, and *Pfcrt*) and the *Pfk13* gene associated with artemisinin delayed parasite clearance, were analysed. To estimate the multiplicity of infection (MOI), and perform *P. falciparum* and *P. vivax* population genetic analyses, the parasites were genotyped by using eight standardized microsatellite loci.

**Results:**

The *P. falciparum* parasites are still harbouring drug-resistant mutations in *Pfdhfr*, *Pfdhps*, and *Pfcrt*. However, there was a decrease in the frequency of highly resistant *Pfdhps* alleles. Mutations associated with artemisinin delayed parasite clearance in the *Pfk13* gene were not found. Consistent with the increase in transmission, polyclonal infections raised from 1.9% in 2003/2004 to 39% in 2018 in *P. falciparum* and from 16.3 to 68% in *P. vivax*. There is also a decrease in linkage disequilibrium. Bayesian clustering yields two populations linked to the time of sampling, showing that the parasite populations temporarily changed. However, the samples from 2003/2004 and 2018 have several alleles per locus in common without sharing multi-locus genotypes.

**Conclusions:**

The frequency of mutations linked with drug resistance in *P. falciparum* shows only changes in *Pfdhps*. Observations presented here are consistent with an increase in transmission from the previously circulating parasites. Following populations longitudinally, using molecular surveillance, provides valuable information in cases such as Venezuela with a fluid malaria situation that is affecting the regional goals toward elimination.

## Background

Although the global malaria burden has been declining for almost two decades [1], Latin America is an exception to this trend. Malaria incidence dropped in the region from 2000 to 2015, resulting in a 61.2% reduction in cases. However, it started to increase from 2015 [1] with Brazil, Nicaragua, and Venezuela driving this surge. During 2018, due to the worsening of the economic and political crisis, Venezuelan cases dramatically increased, accounting for 51% of the region reported cases, displacing Brazil, which reported 23% [1].

Like in other South-American countries [2–4], *Plasmodium vivax* accounts for nearly 77% of Venezuela reported cases followed by *Plasmodium falciparum* (17%), mixed *P. vivax/P. falciparum* infections (5%), and *Plasmodium malariae* with less than 1% [1]. Historically, malaria incidence remained relatively focalized in the endemic areas located in the Bolívar State (bordering Brazil) until 2014 (70–80% of reported cases in the country). An economic and political crisis fueled a flow of migrants to gold mines in Bolívar State, boosting incidence locally, followed by a spillover to other areas within Venezuela, and across international borders [5]. Furthermore, the connectivity among the countries that form the Guiana Shield (Guyana, French Guiana, Suriname, together with parts of Venezuela, and Brazil) [6] has the potential of increasing the regional impact of the Venezuelan situation.

Regional connectivity may not only affect malaria in the region in terms of imported cases but also because the local epidemiology in the area has allowed for the independent emergence of *P. falciparum* mutations linked to anti-malarial drug resistance [6–13]. Thus, such emergent drug-resistant genotypes are at risk of spilling over the region. Hence, to provide must needed baseline information, the genetic makeup of the *P. vivax* and *P. falciparum* populations circulating in Bolívar State, Venezuela was described.

The premise is that a dramatic increase in incidence, as the one observed in Venezuela, should lead to changes in the parasite population in terms of simple metrics, particularly multiplicity of infection (MOI) and linkage disequilibrium (LD) [14]. Also, a population expansion may affect the frequency and dispersion of mutations linked to anti-malarial drugs [14, 15]. Thus, this study compares the population genetic structure and MOI in a population of the Bolívar State between samples collected in 2003/2004, before the ongoing crisis, with samples collected in 2018. All these samples were part of the malaria surveillance programme. In particular, samples from Tumeremo, the capital of the municipality of Sifontes, and surrounding villages in that municipality, were analysed. These human settlements act as a catchment area to those migrating to Brazil and/or working in gold mines.

The hypothesis was that the average multiplicity of infection should increase, and linkage disequilibrium decrease as a result of the sustained demographic expansions of both major malarial parasites. Frequency of mutations were also compared to previously used anti-malarial drugs in *P. falciparum* and interrogated those samples for mutations liked to the artemisinin delayed parasite clearance in the *Pfk13* propeller.

## Methods

### Study sites, sample collection, and DNA isolation

Bolívar is the largest Venezuelan state (242,801 km^2^), it is in the southeastern part of the country bordering Brazil and Guyana. Bolívar accounts for approximately 70% of the Venezuelan cases, mainly among miners and indigenous groups. The state malaria cases are clustered in the Sifontes municipality, an area where gold mining is the major economic activity [5, 12]. Between June 2018 and August 2018, as part of a surveillance study, a total of 182 symptomatic volunteers infected with *Plasmodium* spp. were passively recruited when visiting the health posts for malaria diagnosis. All patients with confirmed malaria infection by microscopy (age range = 13–73 years) were invited to provide a blood sample for further characterization of the parasite, and they received verbal and written explanations about the study. Then, a signed informed consent (IC) form was requested for those patients that agreed to participate. For further molecular analyses to confirm diagnostic and other clinical aspects, a total of 8 mL of blood was collected by venipuncture from every patient, of which 5 mL were stored in tubes containing EDTA and in Whatman™ 903 Protein Saver Cards (GE Healthcare Life Sciences, UK). Malaria infection was determined by microscopic examination of Giemsa-stained thick blood smears (TBS). The study protocol was approved by the Ethics Committee “*Complejo Hospitalario Universitario Ruiz y Páez*.”

To estimate the frequency of drug-resistant mutations in *P. falciparum* and other analyses in both parasites (*P. falciparum* and *P. vivax*), genomic DNA was extracted from anonymized whole blood samples using QIAamp DNA Micro Kit (Qiagen, GmbH, Hilden, Germany).

### Drug resistance genes and *Pfk13* gene

A subset of 50 samples were genotyped for *P. falciparum* mutations at: (1) dihydropteroate synthase (*Pfdhps*) codons 436, 437, 540, 581, and 613; (2) bifunctional dihydrofolate reductase-thymidylate synthase (*Pfdhfr*) codons 50, 51, 59, 108, and 164; and (3) chloroquine resistance transporter (*Pfcrt*) codons 72, 73, 74, 75, 76, and 350. In addition, almost the complete kelch protein *Pfk13* was also amplified (2164 bp out of 2181 bp) for the eighty-eight *P. falciparum* samples gathered in this study. The PCR primer sequences used for each gene were: (1) forward 5′-AAC CTA AAC GTG CTG TTC AA-3′ and reverse 5′-AAT TGT GTG ATT TGT CCA CAA-3′ for *Pfdhps*; (2) forward 5′-ATG ATG GAA CAA GTC TGC G-3′ and reverse 5′-GAA TTC TTC TAC TTG TAG GAT C-3′ for *Pfdhfr*; and (3) forward 5′- TTA CAT ATA ACA AAA TGA AAT TCG C-3′ and reverse 5′-TAT TGT GTA ATA ATT GAA TCG ACG-3′ for *Pfcrt*. In the case of *Pfk13* gene, 88 samples were successfully amplified by nested PCR using external primers forward 5′- GAA TTT TTC TAT NAC RTA YGA DAG-3′ and reverse 5′-ATT TGC TAT TAR NAC NGA RTG NCC-3′; and internal primers forward 5′-ATG ACG TAT GAD AGR GAR TCN G-3′ and reverse 5′-AAT CTG GGA ACT AAT ARD GRD GG-3′ [16].

For *Pfdhps, Pfdhfr*, *Pfcrt*, and *Pfk13* genes, PCR amplifications were carried out in 50 µl volume reaction using 5 µl of total genomic DNA, 2.5 mM MgCl_2_, 1X PCR buffer, 1.25 mM of each deoxynucleoside triphosphate, 0.4 mM of each primer, and 0.03 U/µl AmpliTaq polymerase (Applied Biosystems, Roche-USA). The PCR conditions for *Pfdhps* were a partial denaturation at 95 °C for 7 min, and 40 cycles with 30 s at 95 °C, 30 s at 50 °C and 1 min extension at 68 °C, and a final extension of 5 min at 68 °C in the last cycle. For *Pfdhfr*, the conditions were a partial denaturation at 95 °C for 7 min, and 40 cycles with 1 min at 95 °C, 1 min at 54 °C and 2 min extension at 72 °C, and a final extension of 10 min at 72 °C in the last cycle. For *Pfk13* gene, the conditions for the primary PCR were a partial denaturation at 94 °C for 4 min, and 36 cycles with 1 min at 94 °C, 1 min at 53 °C and 2 min extension at 72 °C, and a final extension of 10 min at 72 °C in the last cycle. The same PCR conditions were used for the nested PCR but with 56 °C for the annealing temperature. In the case of *Pfcrt*, PCR reactions were carried out in 50 μl using TaKaRa LA Taq Polymerase (TaKaRa Mirus Bio Inc.) following manufacturers’ directions. Amplification conditions for the PCR were a partial denaturation at 94 °C for 1 min and 30 cycles with 30 s at 94 °C and 5 min at 60 °C, followed by a final extension of 10 min at 72 °C. Standard *P. falciparum* and *P. vivax* DNA positive and negative (nuclease-free dH2O) controls were included in each batch of PCR. Then, PCR products (50 μl) were excised from the gel and purified using QIAquick^®^ Gel extraction kit (Qiagen, GmbH, Hilden, Germany). Both strands for PCR products were directly sequenced using an Applied Biosystems 3730 capillary sequencer. After careful inspections of each electropherogram, mutations associated with drug resistance (*Pfdhps, Pfdhfr*, and *Pfcrt* genes) or artemisinin delayed parasite clearance resistance were recorded. Sequences obtained in this study for kelch protein *Pfk13* were deposited in GenBank under the accession numbers MT120216 to MT120303.

An alignment with 1099 complete *Pfk13* sequences was performed to explore the genetic relationships among the Venezuelan alleles with those reported worldwide. The alignment included 65 *Pfk13* complete gene sequences obtained here for Venezuelan samples, as well as those available in PlasmoDB and NCBI databases (N = 1034). Then, a median-joining network was estimated by using Network v4.6.1.0 (Fluxus Technologies 2011). Transversions were set equal to transitions, and the epsilon parameter set equal to 0 with only one round of star contraction, which collapses star-like structures in the network into single nodes. The total number of sites included in this analysis, excluding gaps or missing data, were 2155 bp out of 2,181 bp using the *P. falciparum* 3D7 strain (PF3D7_1343700) as a reference. Estimates of haplotype diversity and Tajima’s D test were obtained using DnaSP software, version 6 [17]. Also, evidence of natural selection was explored. In particular, the average number of synonymous substitutions per synonymous site (dS) and the average number of nonsynonymous substitutions per nonsynonymous site (dN) were calculated between all pairs of sequences from the Venezuelan samples (2018). This analysis was carried out using the Nei-Gojobori method with the Jukes and Cantor corrections, as implemented in the MEGA v7 software [18]. The null hypothesis tested was that the observed polymorphism is not under selection (dS = dN). The difference between dS and dN with its standard error (estimated using bootstrap analysis with 1000 pseudo-replicates) was calculated, and a two-tailed codon-based Z-test tested for significance on the difference between dS and dN [19].

### Microsatellite (STRs) genotyping

Genotyping was performed using fluorescently labeled PCR primers for a set of eight standardized microsatellites for *P. falciparum* (POLYa, TAA60, ARA2, Pfg377, TAA109, TAA81, TAA42, and PfPK2 [20] and nine for *P. vivax* (MS1, MS2, MS5, MS6, MS8, MS10, MS15 [21], 14.185, and 2.21, [22]). PCRs were performed in 15 μL reactions with 2 μL of total genomic DNA, 0.25 mM of each primer, and 7.5 μL of PCR Master Mix (Promega, USA) (it contains 0.05 U/μL of Taq DNA polymerase, 2X reaction buffer, 0.4 mM each dNTP, and 3 mM MgCl2; Promega, USA). *P. falciparum* and *P. vivax* DNA positive samples and negative (nuclease-free dH2O) controls were included in each batch of PCR, respectively. Amplification conditions for PCRs were reported elsewhere, depending on the set of primers [20–22]. Fluorescently labelled PCR products were separated on an Applied Biosystems 3730 capillary sequencer and scored using GeneMarker v2.6.7 (SoftGenetics LLC). All alleles were scored at a given locus if minor peaks were more than one-third the height of the predominant peak. The finding of one or more additional alleles at any locus was interpreted as a multiple (polyclonal) infection with two or more haploid genotypes in the same isolate (transmitted by one or several mosquitoes). A sample with a single infection was considered if it had only one allele per locus at all the genotyped loci. Missing data (no amplification) were reported by locus but not considered for defining multilocus genotypes.

### The multiplicity of infection (MOI) and allele frequencies

MOI was defined as the average number of distinct parasite (*P. falciparum* or *P. vivax*) genotypes concurrently infecting a patient. Thus, average MOI in these *P. falciparum* and *P. vivax* populations was estimated (per loci—ignoring missing data-and on average per loci/year) as the average number of super-infections (neglecting co-infections) using a maximum-likelihood (ML) method that allows estimating profile-likelihood confidence intervals [23, 24]. Then, MOI and allele frequencies were calculated using the R-script provided in [24]. In the case of allele frequencies, they were estimated for each locus using ML method [23].

### *Plasmodium falciparum* and *P. vivax* population genetic analysis

A suite of approaches was used to characterize the genetic variation in the circulating *P. falciparum* and *P. vivax* and the differentiation of the parasite populations between 2003/2004 and 2018. In particular, the microsatellite data gathered in this investigation were compared against previous data reported for the same location in 2003/2004 [25]. The genetic diversity within each parasite population was estimated using a series of summary statistics implemented in the Haplotype Analysis software v1.05 [26]. For both parasites (*P. falciparum* and *P. vivax*), the number of different sampled multilocus genotypes (SMG), the number of unique genotypes (G), the number of private genotypes (PG), and the Nei’s index of genetic diversity (He) were estimated [27] on all the multi-locus genotypes that were unambiguously identified. *He* was estimated as

$$ He = \left[ {n/\left( {n - 1} \right)} \right]\left[ {1 - \sum\limits_{i = 1}^{L} {pi^{2} } } \right] $$

where *n* is obtained by taking the sum of identifiable genotypes (phased) overall samples, and *p*_*i*_ is the relative frequency of the *i*-th haplotype (*i *= 1, …, L) in all sampled haplotypes. *He* gives the average probability that a pair of alleles randomly selected from the population is different. Complex infections with differences at more than two loci were not included in this analysis because the haploid genotypes could not be inferred. *He* was also calculated per locus. In this case, *p*_*i*_ is the frequency of allele *i*, and *He* is the average probability that a pair of alleles randomly selected from the population is different. Non-parametric bias-corrected and accelerated bootstrap confidence intervals were estimated based on 1000 bootstrap replications using the jackknife estimate for the acceleration factor, as described in [28].

Two methods were used to identify the parasite clusters (*P. falciparum* and *P. vivax*) that circulated in Bolivar State, (i) principal component analyses (PCA) that do not use explicit admixture model (but that is not assumption-free), and (ii) a Bayesian model-based clustering algorithm that considers admixture as implemented in the Structure v2.3.4 software [29]. PCAs were estimated for both population parasites (*P. falciparum* and *P. vivax*) in R-script. To include samples with missing data and samples with multiple infections, for each PCA, alleles at each locus were coded as 0–1 variable, indicating the absence and presence of the allele. Hence, the number 0–1 variables for each locus reflects the number of alleles at that locus. This allowed exploring the effect of potential amplification errors without eliminating samples.

For the Bayesian clustering approach, which assigns genotypes to K populations or clusters characterized by a set of allele frequencies at each locus, the observed genetic diversity was evaluated under different K values (K = 1 to 12), and each K value was run independently 15 times with a burn-in period of 100,000 iterations followed by 100,000 iterations. The admixture model that allows for the presence of individuals with ancestry in two or more of the K populations was used in all the analyses [29]. The posterior probability for each number of populations or clusters (K) was computed, and the K-value that better explains the genetic data was an estimate of the number of parasites circulating clusters. Delta K values were computed using Structure Harvester [30] and then, CLUMPP (Cluster Matching and Permutation Program) was used to facilitate the interpretation of population-genetic clustering results [31]. Finally, distruct v1.1 was used to display the results graphically [32]. For this analysis, complex infections with differences at more than two loci were not included.

To evaluate the differentiation in time between the parasite populations, 2003/2004 vs. 2018, normalized fixation indexes (*Fst*) were estimated; their significance was assessed using a randomization test. A limitation in this analysis is that haploid genotypes cannot be inferred on samples with multiple alleles at two or more loci, so such samples were excluded. Considering that these are neutral physically unlinked loci, measurements of linkage disequilibrium (LD) between pair of loci were also estimated to detect potential clonal expansions of both parasite populations. Conditional asymmetric linkage disequilibrium (ALD) measurements were used since they consider differences in the number of alleles in each pair of loci (many pairs of loci have a different number of alleles) [33]. ALD estimates go from 0 to 1 with 0 implying total independence and 1 complete linkage. ALD requires frequency estimates of the two-locus haplotypes observed at each pair of loci under consideration. Thus, two-locus haplotypes were inferred between all pairs of loci, and their frequencies estimated using the ML method of [23] using the R-script provided in [24]. If there were multiple alleles at two loci under consideration for a given pair of samples, their haplotypes could not be phased, and just that comparison was disregarded for that pair of samples.

Finally, population genetic analyses were complemented by inferring the haplotype genealogies for *P. falciparum* and *P. vivax* populations contrasting the data obtained from 2003/2004 against those from 2018. These haplotype genealogies were estimated on eight microsatellites for *P. falciparum* and nine for *P. vivax*, using the Global Optimal eBURST algorithm [34], as implemented in PHYLOViZ [34]. These analyses only included single infections and complex infections with differences at only one locus, given that the haploid genotypes can be inferred. A Minimum Spanning Tree-like (MST) structure was drawn to cluster the 198 sequence types (STs) for *P. falciparum* and 159 for *P. vivax* into a clonal complex (CC) based on their multilocus genotypes by using an extension of the goeBURST rules up to n-locus-variants-level (nLV, where n equals to the number of loci in these datasets: eight for *P. falciparum* and nine for *P. vivax* respectively). In addition, to better explore the resulting tree and the relationships between genotypes, an NLV graph was obtained using a different operation that modifies the default characteristics of the MST. This kind of NLV graph easily identifies sets of closely related nodes by relaxing the MST construction restriction, allowing the display of all possible links up to a specific threshold.

## Results

In this study, 88 patients were infected with *P. falciparum* and 94 with *P. vivax*. Out of the 88 infected with *P. falciparum,* 19 were co-infected with *P. vivax* (mixed infections) detected by PCR.

### Drug resistance genes

No multiple-strain infections were detected in the electropherograms. In 2018, mutations associated with sulfadoxine-pyrimethamine (SP) resistance in *Pfdhfr* and *Pfdhps* genes revealed four multilocus genotypes: (1) *Pfdhfr* (50R/51I/108 N) linked to *Pfdhps* (437G/540E/581G) present in 58.1% of the samples (hereinafter referred to as a sextuple mutant), (2) *Pfdhfr* (50R/51I/108 N) linked to *Pfdhps* (437G/581G) in 32.6% of samples, (3) *Pfdhfr* (50R/51I/108 N) linked to *Pfdhps* (437G) in 7% of the samples, and (4) *Pfdhfr* (51I) linked to *Pfdhps* (437G) present only in 2.3% of the samples (only one patient) (Table 1). These results contrast with a previous report where the sextuple mutant was present in 90.7% of the samples, and a quadruple mutant *Pfdhfr* (51I/108 N) linked to *Pfdhps* (437G/581G) was present in 9.3% of the samples [8]. Although, in 2003/2004 two synonymous of *Pfcrt* chloroquine resistance alleles, S_tct_VMNT (91%) and S_agt_VMNT (9%) were circulating in the population [9], in 2018 only the allele S_tct_VMNT was found in the set of samples used (Table 1).

**Table 1:** Percentage of *P. falciparum* samples with mutations associated with SP and chloroquine resistance in *Pfdhps*, *Pfdhfr* and *Pfcrt* genes in 2018

Gene	N	Mutants		%
*dhps*	28	Triple	A437**G**, K540**E**, A581**G**	56
	17	Double	A437**G**, A581**G**	34
	5	Single	A437**G**	10
*dhfr*	42	Triple	C50**R**, N51**I**, S108**N**	97.7
	1	Double	N51**I**, S108**N**	2.3
*Pfcrt*	45	Double	C72**S**, K76**T** (**S**_**ctc**_VMN**T**)	100

N total samples successfully amplified (out of 50), mutations associated with SP and chloroquine resistance are shown in bold

### *Pfk13* population analyses

Eighty-eight *Pfk13* sequences were obtained in this study for the group of samples collected in 2018. The propel region was sequenced for all 88 samples to check for mutations associated with artemisinin delayed parasite clearance phenotype. However, only 65 *Pfk13* alleles were sequenced completely and used for the genetic diversity analyses. For this dataset, a very low genetic diversity was found in *Pfk13* gene (π = 0.0005 ± 0.0), with only two haplotypes circulating in the area (Table 2). In addition, the polymorphism measured using the number of polymorphic (segregating) sites had an S value of 1 (Table 2). Indeed, only two sites showed substitutions in the Venezuelan sequence alignment, one was a nonsynonymous substitution outside of the propeller domain (K189T; 55.4%), and the other was a synonymous substitution (V_GTT_636V_GTG_, 1.5%) in the propeller domain (440-680 bp). More important, none mutations associated with the delayed parasite clearance phenotype were found in the Venezuelan samples.

**Table 2:** Polymorphism found in *Pfk13* gene of *P. falciparum* from Venezuela samples from 2018

	π (SE)	DS	dN	dS-dN (SE^a^)	P value (Z-statistic^b^)	S^c^	Tajima’s D (P value)	No. of haplotypes (Hd)	Haplotype (gene) diversity (SD^d^)
Venezuela (N = 65)	0.0007 (0.0003)	0	3E−04	− 0.0003 (0.0003)	0.309 (1.021)	1	1.739, 0.10 > P>0.05	2	0.502 (0.017)
Americas (N = 488)	0.00003 (0.00002)	8E−05	2E−05	0.00006 (0.00008)	0.468 (− 0.728)	2	− 1.260, P > 0.10	3	0.0164 (0.011)
Total (N = 1099)	0.0006 (0.0002)	3E−04	6E−04	− 0.0003 (0.0003)	0.294 (1.053)	120	− 2.62283, P < 0.001	124	0.7031 (0.012)

^a^ Standard error (SE) estimates are from 1000 bootstrap replicates (MEGA7)

^b^ P values from the codon-based Z-test are shown (MEGA7)

^c^ Segregating sites

^d^ Standard Deviations (DnaSP6)

Then, these sequences (N = 65) were analysed together with 1034 *Pfk13* gene sequences from around the globe, including samples from The Americas (Table 2, Additional file 1). Thus, the K189T mutant found in Venezuelan samples was the most predominant in Africa, Brazil, and French Guyana. In contrast, the V_GTT_636V_GTG_ found in one Venezuelan sample was previously reported in two sequences from India and one from Nigeria (Additional file 1). When sequences from Central and South America were compared, there were only two nonsynonymous (G496V and S679P) and four synonymous substitutions (G453G, K503K, G638G, and F673F, Additional file 1) within the propeller domain in the Haitian sequences (N = 103) and all of them in very low frequency (0.9–8.7%). It worth noticing that none of them have been associated yet with the clinical delayed parasite clearance. Interestingly, none substitutions were found in the propeller domain from other countries of The Americas.

**Fig. 1:**
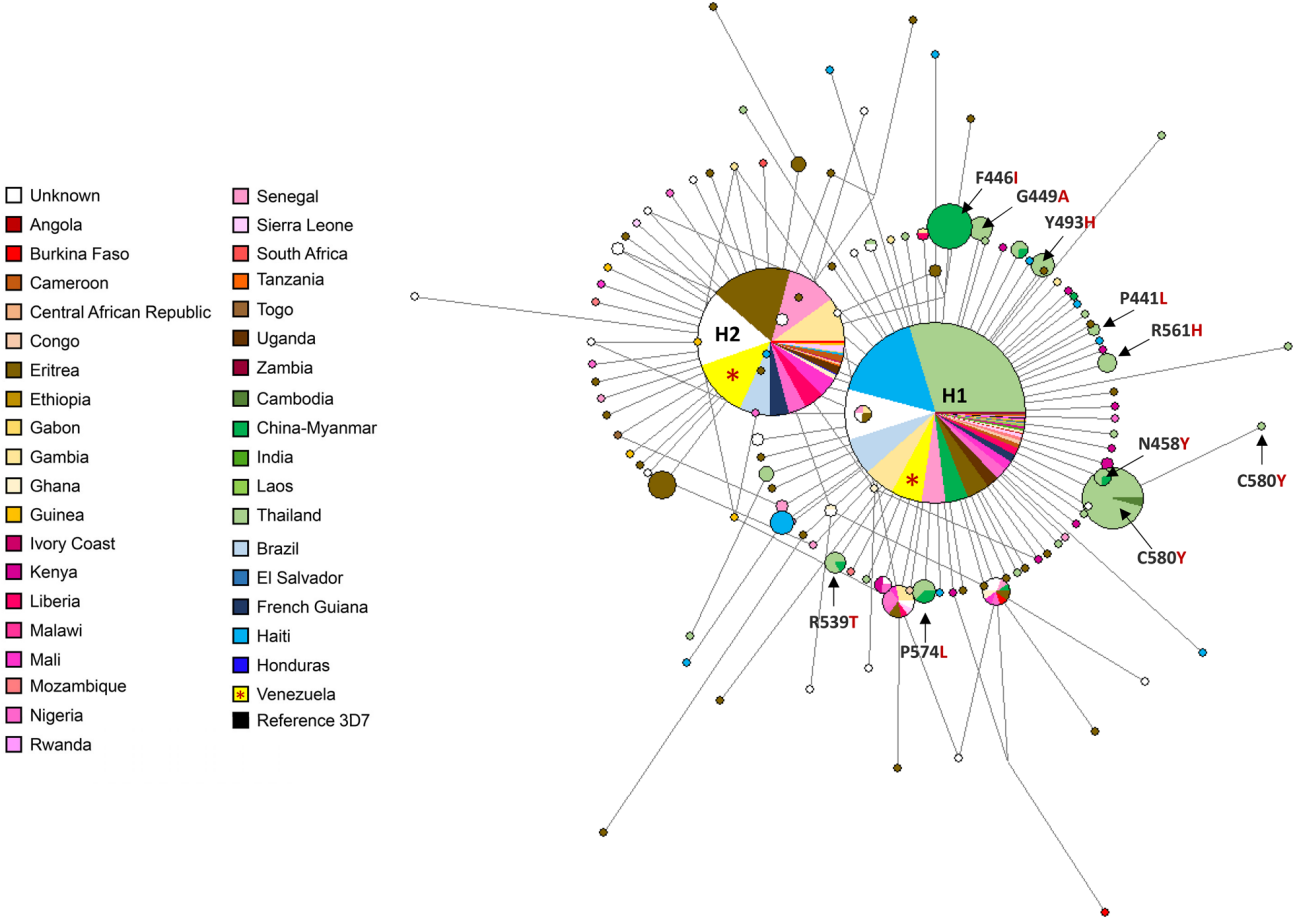
Median-joining network of the *P. falciparum Pfk13* haplotypes using 1099 complete sequences from a global sample. All complete *Pfk13* gene sequences obtained here for Venezuelan *P. falciparum* population samples (n = 65) in 2018, as well as those available in the PlasmoDB and NCBI databases (n = 1034), were included. Colors correspond to a different geographic origin and the branch lengths are proportional to divergence; node sizes are proportional to the total haplotype frequencies. Lines separating haplotypes represent mutational steps. Mutations associated with the delayed parasite clearance phenotype are indicated with arrows. The most frequent haplotypes are indicated as H1 and H2, and the two haplotypes found in Venezuelan samples are shown in yellow and labelled with an “*”

Worldwide, a total of 124 *Pfk13* haplotypes were found with a haplotype (allele) diversity (Hd) of 0.7031 (standard deviation [SD] = 0.0117) (Table 2). These results are similar to those recently reported by Pacheco et al. [16]. Median-joining network for the *Pfk13* gene, estimated using Network software (Fluxus Technologies, 2011), showed two star-like shapes with at least two most predominant and common alleles H1 and H2 (Fig. 1) with a broad distribution (Asia, Africa, and South America). One of the alleles (H1, n = 525, 47.8%) is mostly distributed in Asia, and the other (H2, n = 282, 25.7%) in Africa. Interestingly, both haplotypes contain sequences from Venezuela and Brazil. Specifically, for Venezuelan samples, out of the 65 complete sequences, 36 (55.4%) corresponded to the H1 and 29 (44.6%) to the H2, which contains the K189T mutant (Fig. 1). A third haplotype (H3, n = 50, 4.5%), in terms of frequency, is only found in Thailand and contains the C580Y mutation found in the SEA region (Fig. 1). Nevertheless, most of the haplotypes were restricted to single countries (Fig. 1).

### The multiplicity of infection and allele frequencies

**Fig. 2:**
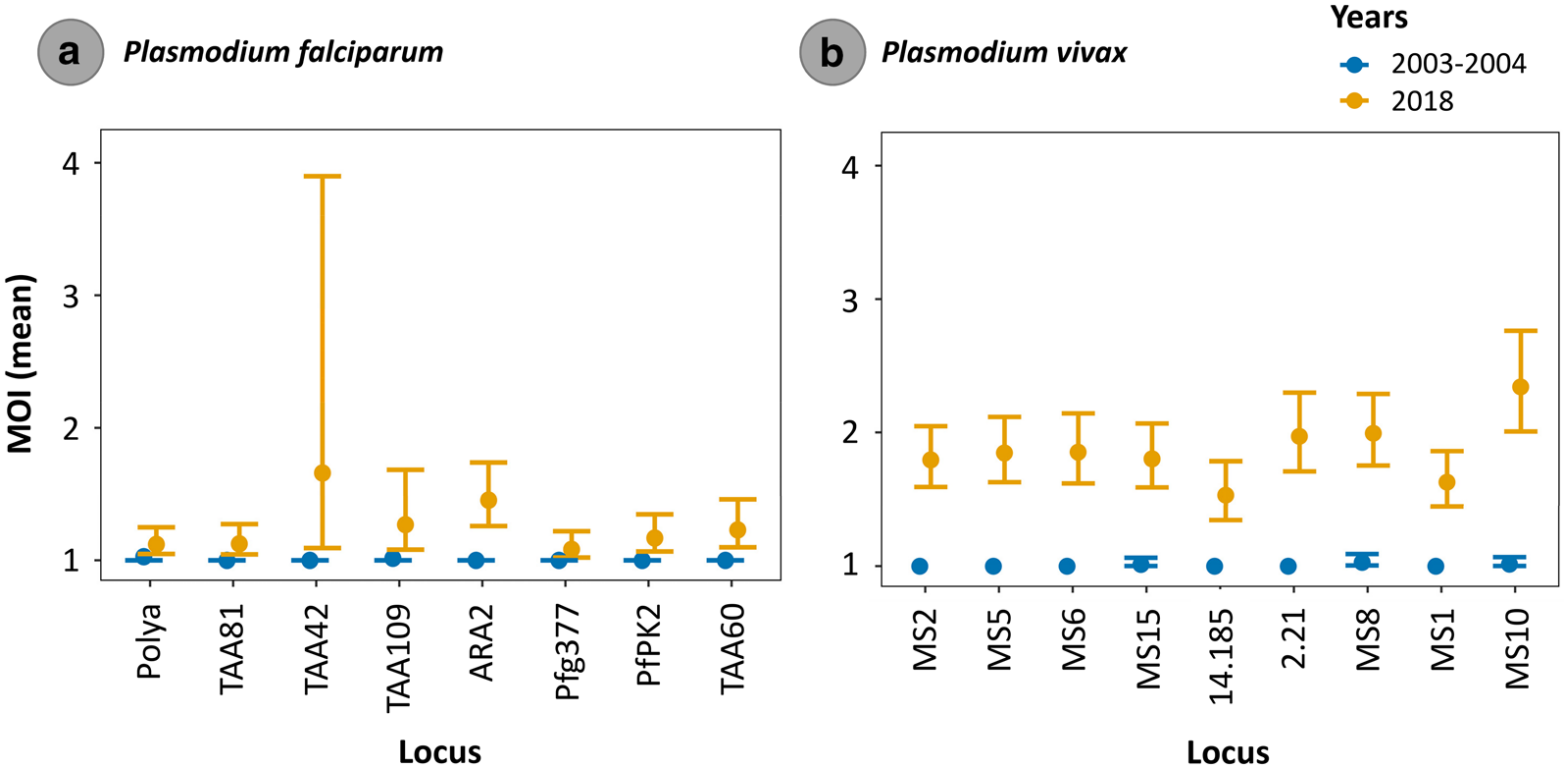
Mean Multiplicity of Infection (MOI) with its 95% profile-likelihood confidence intervals per locus for *P. falciparum* (**a**) and *P. vivax* (**b**) populations sampled in 2003/2004 and 2018

Overall, for *P. falciparum* and *P. vivax* infections, the prevalence of multiple infections showed a substantial increase from 2003/2004 to 2018. Many of those *P. falciparum* and *P. vivax* multiple infections were the result of having more than one allele in one or two loci, especially in *P. vivax* (Table 3), so the lineage-specific genotypes were only inferred for those samples with more than one allele at only one locus. In 2018, among the *P. falciparum* cases, 39% of the 77 samples successfully genotyped (using microsatellites) had infections with more than one lineage in at least one locus. In contrast, 68% out of 94 *P. vivax* samples were found with multiclonal infections (Table 3). This difference translated into a slightly higher MOI in *P. vivax* (> 1.5 vs. < 1.5, Fig. 2) with overall more complex infections (several loci with up to three alleles). In addition, the estimated MOI parameter showed that the mean MOI also substantially increases for each microsatellite marker from 2003/2004 to 2018 in both parasite populations, especially for *P. vivax* (Fig. 2b). This increase was statistically significant for both parasite species (Additional file 2).

**Table 3:** Observable variation within infections for *P. falciparum* and *P. vivax* Venezuelan populations sampled in 2003/2004 and 2018

	2003/2004		2018	
	No.	%	No.	%
*P. falciparum*				
Total human samples	104		77	
Total single infection	102	98.1	47	61.0
Total multiclonal infections	*2*	*1.9*	*30*	*39.0*
Total samples with 2 alleles/at least one locus	2		29	
Total samples with 2 alleles/≥ 2 loci	0		11	
Total samples with 3 alleles/at least one locus	0		1	
Total samples with 3 alleles/≥ 2 loci	0		1	
*P. vivax*				
Total human samples	104		94	
Total single infection	87	83.7	30	32.0
Total multiclonal infections	*17*	*16.3*	*64*	*68.0*
Total samples with 2 alleles/at least one locus	14		35	
Total samples with 2 alleles/≥ 2 loci	9		18	
Total samples with 3 alleles/at least one locus	2		23	
Total samples with 3 alleles/≥ 2 loci	2		22	
Total samples with 4 alleles/at least one locus	0		3	
Total samples with 4 alleles/≥ 2 loci	0		3	
Total samples with 5 alleles/at least one locus	0		3	
Total samples with 5 alleles/≥ 2 loci	0		3	

Values in italics are for comparison between 2003/2004 and 2018

### Population genetic analysis

**Fig. 3:**
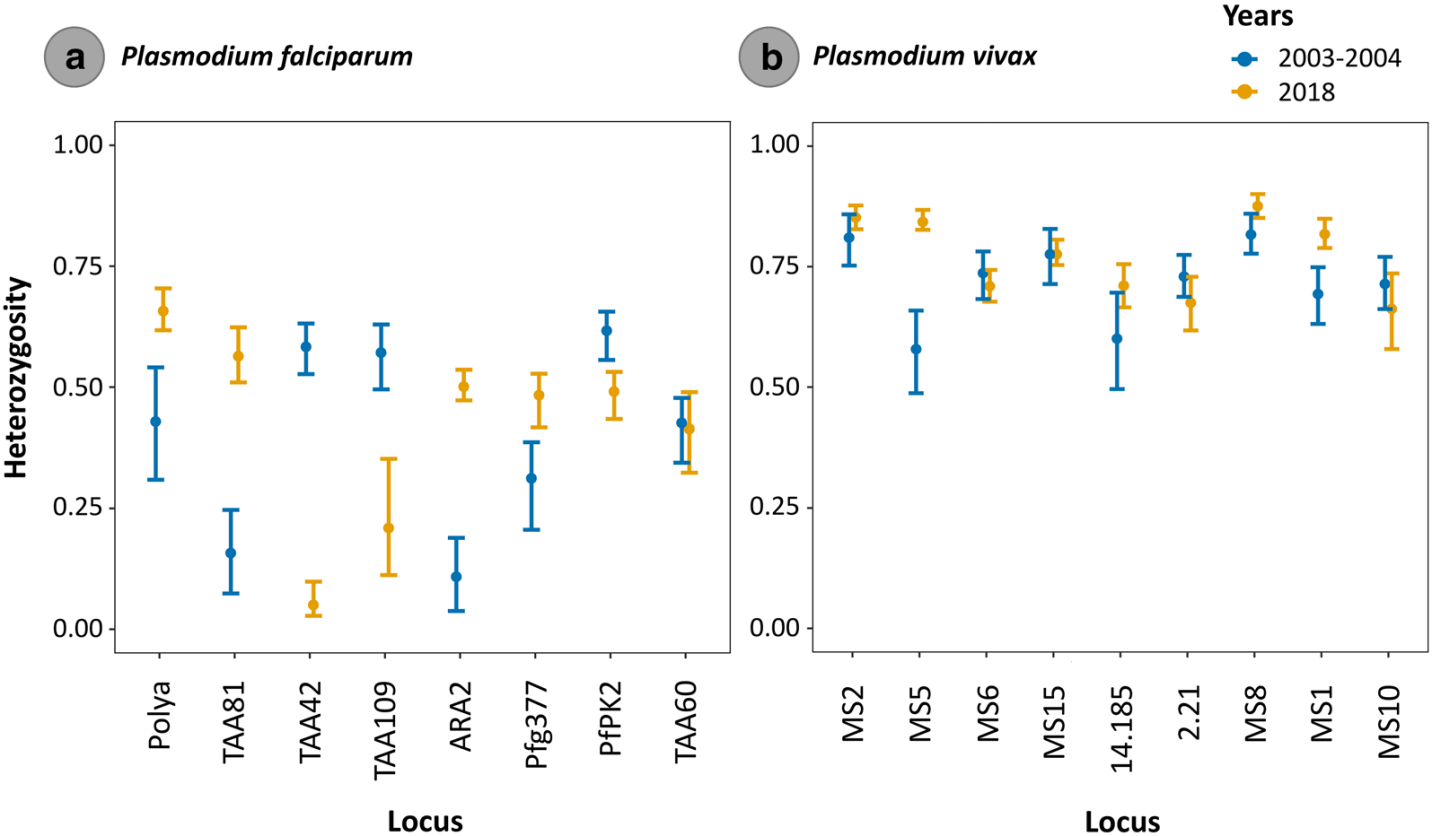
*Plasmodium falciparum* (**a**) and *P. vivax* (**b**) heterozygosity and its 95% bias-corrected and accelerated non-parametric bootstrap confidence intervals. Parasite heterozygosity is shown per locus and years (2003/2004 and 2018)

Using those samples for which their haplotypes could be inferred, the number of sampled multilocus genotypes (SMG) from the human specimens, the number of distinct genotypes (G), the number of private genotypes (PG), and the Nei’s index of genetic diversity (He) were estimated for each population using Haplotype Analysis software v1.05 and are shown in Table 4. Interestingly, mean genetic diversity was high and similar in both parasites (*P. falciparum*-mean He: 0.939 and *P. vivax*-mean He = 0.938), and none of the *P. falciparum* and *P. vivax* genotypes found in 2003/2004 were recovered in 2018. *F*st values were significant and relatively high for both parasites (Table 4). When *He* was also calculated per locus, there was some overall tendency to increase heterozygosity but not for each locus in the case of *P. falciparum* (only 4 loci, Fig. 3a and Additional file 3). However, for *P. vivax*, there were high levels of heterozygosity in 2003/2004 and 2018. There was an overall tendency to increase heterozygosity in 2018 but not for each locus (only 5 loci, see Fig. 3b). The number of alleles per locus was similar between the sampled years for both parasite populations (Additional files 4 and 5), but with a tendency to increase the number of alleles in 2018 for *P. vivax* with a greater number of low-frequency-variants.

**Table 4:** Diversity of multilocus genotypes per parasite population and sampled years (2003/2004 and 2018)

Years	SMG	G	PG	He	*F*st
*P. falciparum*					
2003/2004	84	22	22	0.896	0.036
2018	66	43	43	0.982	0.033
Mean		32.5	30.5	0.939	0.035
*P. vivax*					
2003/2004	90	37	37	0.877	0.043
2018	46	45	45	0.999	0.038
Mean		41	41	0.938	0.041

**Fig. 4:**
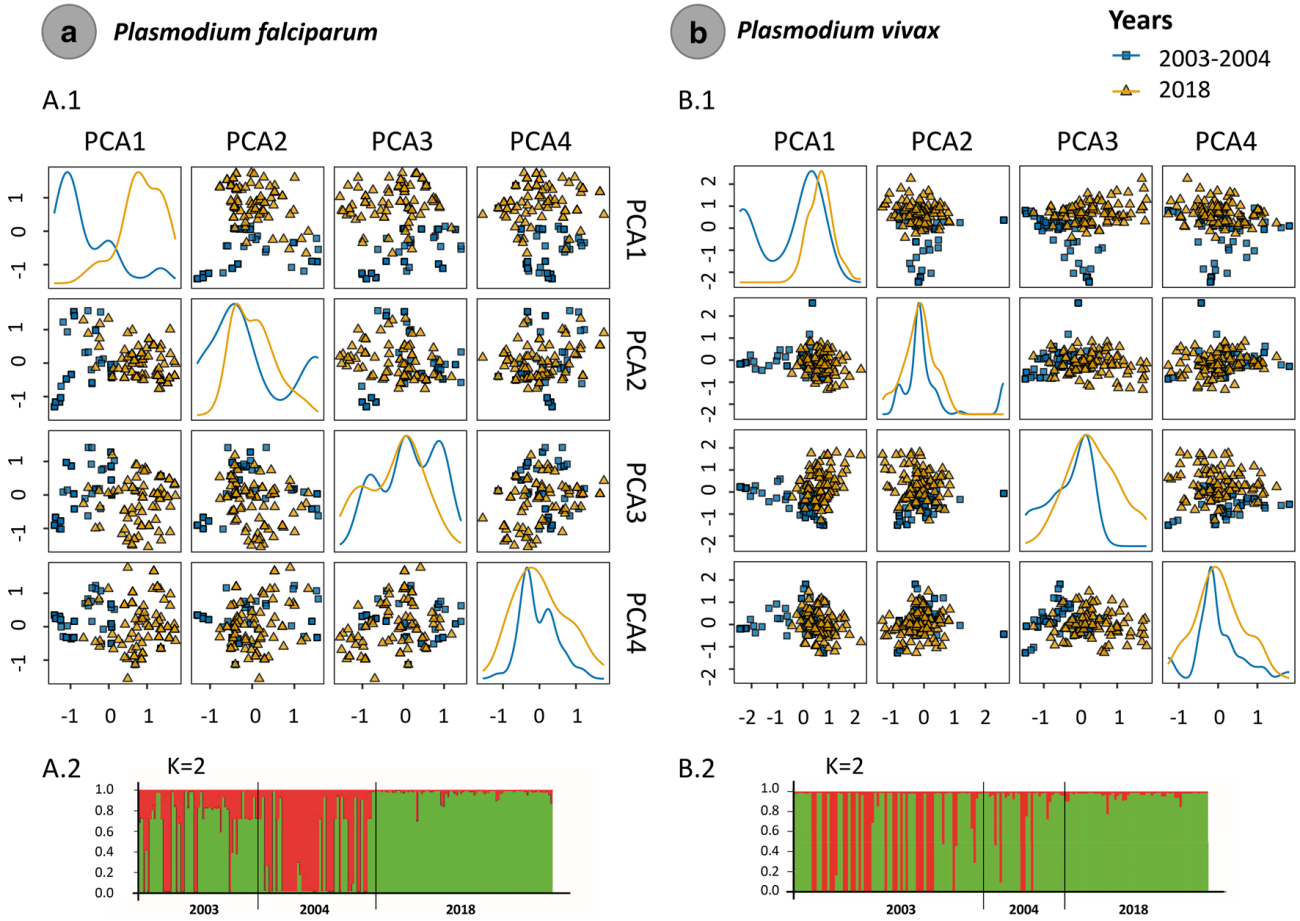
Principal component analysis (PCA) for *P. falciparum* (**a**.1) and *P. vivax* (**b**.1) populations sampled in 2003/2004 and 2018. *Plasmodium falciparum* (**a**.2) and *P. vivax* (**b**.2) population structure (2003/2004 and 2018)

In the case of the PCA analyses, for *P. falciparum* dataset, 64.2% of the variance between the sampled years was explained by the first 4 components (Fig. 4a, Additional file 6) and the first PC component separates both indicating a shift between the two time points (2003/2004 and 2018). In contrast, the four PCs explained around 34.5% of the variance between the two *P. vivax* dataset (Fig. 4b, Additional file 6). By analysing the posterior probabilities for each clustering from K = 1 to 12, the clustering obtained with K = 2 showed the highest Delta K values (Delta K = mean (|L”(K)|)/sd(L(K))) for both *Plasmodium* species. Thus, the Bayesian clustering using the Structure v2.3.4 software [29] yield two populations (K = 2) for *P. falciparum* and *P. vivax* and linked to the time of sampling showing that both parasites populations diverged in time (Fig. 4).

**Fig. 5:**
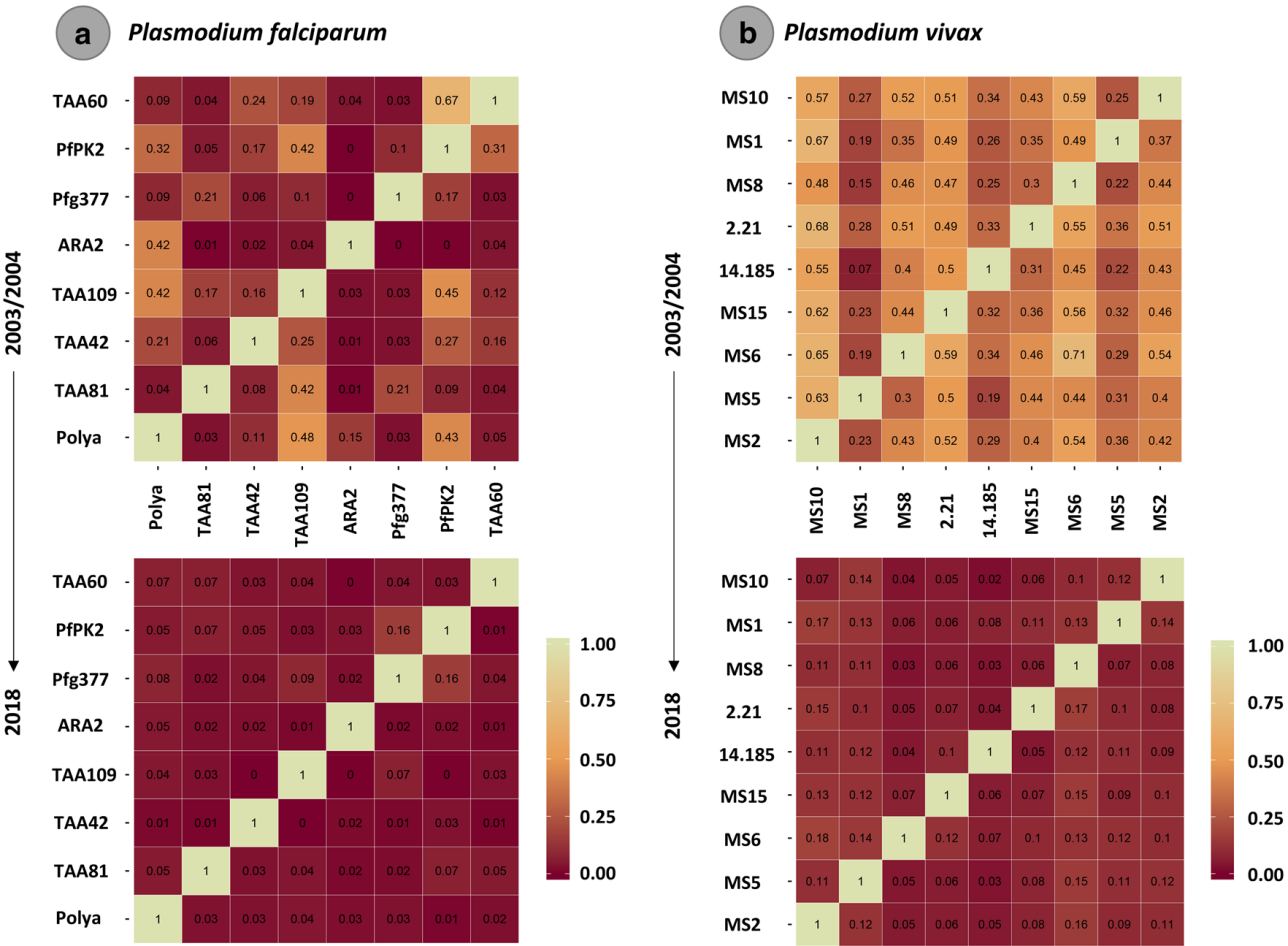
Measurements of conditional asymmetric linkage disequilibrium (ALD) per pair of microsatellite loci for *P. falciparum* (**a**) and *P. vivax* (**b**) populations sampled in 2003/2004 and 2018

**Fig. 6:**
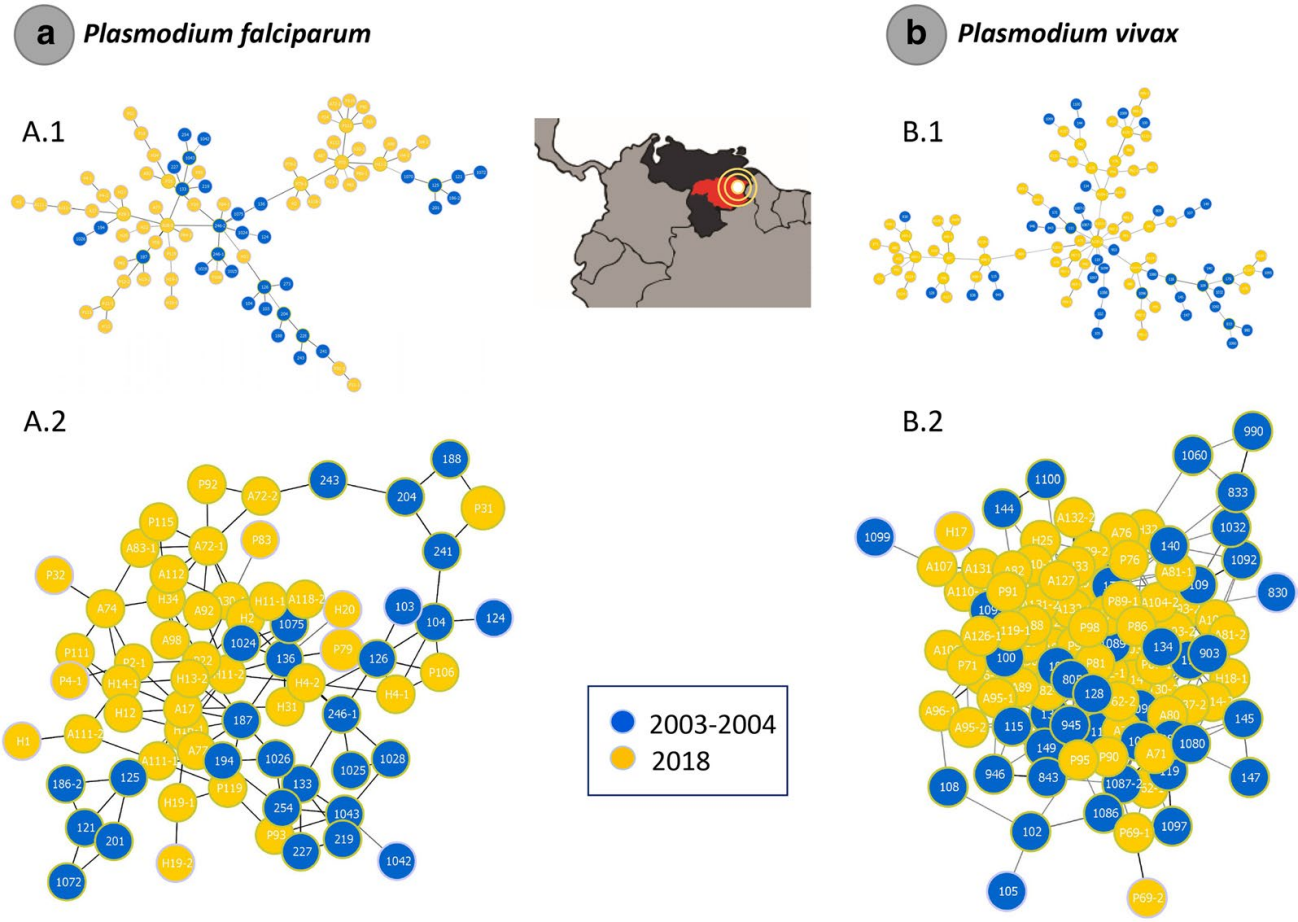
Minimum spanning tree and N Locus Variant (NLV) graph for *P. falciparum* (**a**.1, **a**.2 respectively) and *P. vivax* (**b**.1, **b**.2 respectively) constructed using goeBURST. The trees depict the relationships among parasites sequence types (ST) at the nLV level (where n equals the number of loci in these datasets: eight and nine respectively). Each ST is represented by a circle, and the size of the circle is logarithmically proportional to the number of samples with that particular ST. The color of each circle represents the sampled year

Although the asymmetric linkage disequilibrium (ALD) estimated for *P. falciparum* was stronger for 2003/2004 than in 2018, the values per locus were low for both sampled periods (Fig. 5a). However, for *P. vivax*, the pattern was slightly different, with strong ALD in 2003/2004 and almost absent in 2018 (Fig. 5b). Low ALD of physically unlinked loci, as used here, is contrary to the expectations under a bottleneck or clonal expansion scenarios. Lastly, the haplotype genealogies inferred for *P. falciparum* (Fig. 6a.1) and *P. vivax* (Fig. 6b.1) populations and the NLV graph (Fig. 6a2, b2) showed no clear boundaries between the sampled periods.

## Discussion

The increase in malaria cases in Venezuela, and the subsequent mass migration across international borders, is likely the most important event affecting the goal toward malaria elimination in Latin-America [5, 13, 35]. It is worth noting that the long-term impacts of the current situation in Venezuela, in the context of cross-border malaria, are still poorly understood. Most reports focus on counting the number of imported cases [5, 35]. Yet, data are being collected in other aspects, such as the prevalence of mutations linked to drug resistance in *P. falciparum* [12, 13]. Here, baseline data on the circulating parasites found in Bolívar State in 2018, the epicentre of the current malaria epidemic affecting Venezuela, were described. Although there are other factors, the surge of unplanned gold mining activities with limited access to effective anti-malarial treatment is considered the leading cause that drove the increase in malaria transmission observed in the Bolivar state [4–6].

Bearing in mind the limitations of the sample size, it is worth noting that 21.5% (19 of the 88 *P. falciparum* positive parasites) were mixed infections with *P. vivax* detected when using PCR. These results differ from the 6.8% reported from the same area in a recent study [36], but using different protocols. Thus, it may be possible that the reported national average of mixed infections (5%) is an underestimation. Considering that all those infections were diagnosed as *P. vivax*, inadequate anti-malarial treatment could lead to an increase in “new infections” by *P. falciparum* that were undetected mixed infections treated with chloroquine.

Although SP and chloroquine are no longer used to treat *P. falciparum*, fluctuations in the frequency of mutations linked to resistance were observed in 2018 when compared to 2003/2004. Unlike the situation found in some African countries [37, 38], the lack of *Pfcrt*-wild type in the *P. falciparum* population did not allow the recovery of sensitive chloroquine parasites even when the drug is no longer used to treat falciparum malaria. This scenario was suggested by early studies [8, 9]. The only way that chloroquine-sensitive parasites could re-emerge in this region is via their reintroduction by migration, something that has not been documented, or the emergence of a new mutation that revert to chloroquine sensitivity [10]. It could be speculated that the *P. falciparum* population is still under some chloroquine drug pressure. The drug is commonly used to treat uncomplicated *P. vivax,* and there could be a treatment spillover due to mixed infections or inadequate diagnostic [39]. The fixation of the *Pfcrt* S_tct_VMNT can be explained by genetic drift since it was the most common allele in the region [8, 9].

The situation is different in SP. Like has been reported in Peru [40, 41], there is a decline in the highest resistant alleles. Here, however, that decline was observed on the frequency of the *Pfdhps* triple mutant that confers high resistance to sulfadoxine, one of the SP components. It is also worth noting the presence of a single *Pfdhps* mutant; this allele was not observed in an early study [8]. Thus, the regional changes in malaria treatment seem to have affected at least the frequency of mutations linked to sulfadoxine resistance.

Mutations linked to artemisinin delayed phenotype in *Pfk13* were not found in the 88 isolates genotyped for this gene; a result consistent with a recent study on samples collected on Venezuelan migrants in Brazil [13]. This is an important finding since there is constant movement between Guyana, Brazil, and Venezuela as a result of the illegal exploitation of gold mines [6]. Fortunately, it seems that the *Pfk13* mutations reported in the region [11] remind contained. Still, the circulating *Pfk13* alleles show polymorphism with mutations observed in low frequency in other areas. E.g., the K189T mutant found in Tumeremo has been found in Africa and neighbouring countries such as Brazil and French Guyana. Indeed, the alleles circulating in Venezuela belong to the two major alleles already reported [16]. Such local substructure likely accounts for the positive, but no significant, Tajima’D value reported here (Table 2). The world sample with N = 1099 sequences has a significant and negative Tajima’D consistent with a population expansion or selective sweep, the later less probable simply because this sample of worldwide alleles only has a few of the mutations conferring resistance. The bias on reporting resistant alleles has to do with how the data on *Pfk13* mutations is usually generated [16].

Nevertheless, the worldwide presence of mutations in low frequency in the *Pfk13* gene could be explained, at least in part, by the *P. falciparum* population expansion. This demographic process could make natural selection less efficient to eliminate otherwise semi-deleterious mutations [16]. Such a repertoire of mutations in low frequency may provide the variation for drug-mediated selective pressure to act.

The observed increase in the number of polyclonal infections (multiplicity of infection or MOI) in both parasites between 2003/2004 and 2018 correlates with the overall changes in transmission [14, 42–46]. Although MOI measures the frequency of superinfections and co-infections [23], its relationship with transmission intensity is not linear [42–44]. In particular, comparing MOI between regions that differ in transmission does not seem to be a sound approach if those populations also differ in other epidemiological and historical characteristics, including the genetic diversity/evolutionary history of the circulating parasites [42–44, 46]. Understandably, MOI is also affected by those other factors making it a poor predictor of transmission intensity in such contexts [43–45]. Here, however, the increase in the average MOI appears to be explained by the surge in transmission.

The differences in MOI are interesting also because the genetic diversity in Tumeremo has a moderate increase in both parasites locally. There was observable differentiation when the two different time points are compared, in both *P. falciparum* and *P. vivax*, using PCA and structure analyses indicated temporal structure (Fig. 4). However, such differentiation seems to be explained by recombination and changes in allele frequencies from a common allele pool rather than migration, as indicated by the haplotype network (Fig. 6). One of the factors that indicate this expansion from the original population are the changes in linkage disequilibrium, as it decreases in both parasites when 2018 parasites are compared with those collected in 2003/2004. Thus, as transmission intensifies and the frequency of polyclonal infections increased, also the rate of outcrossing increased, breaking the linkage that originally was sustained by inbreeding when malaria was under control in 2003/2004 among these microsatellites that are physically unlinked. MOI, in addition to changes in transmission, has been linked to disease severity for *P. vivax* in some settings [42]. However, its relationship with clinical disease severity may depend on several factors, including how MOI is measured in terms of loci, sampling, clinical endpoints, and age, among other confounding factors [42–46]. A clinical study should provide some insights into the role of MOI in setting such as Venezuela, where transmission has reached unprecedented high levels. Nevertheless, the observed changes in MOI and linkage disequilibrium are consistent with the use of these metrics to follow transmission intensity longitudinally [45–48].

## Conclusion

The frequency of mutations linked with drug resistance changed in SP, where the highly resistant triple mutants decreased in frequency, particularly in the case of *Pfdhps* gene. These have been observed in other areas in Latin America where SP is no longer used to treat *P. falciparum* malaria. Importantly, no evidence of *Pfk13* mutations associated with artemisinin delayed clearance were found here. Overall, population-level parameters such as the average MOI and linkage disequilibrium are internally consistent and affected by the increase in malaria incidence observed in Venezuela. These findings indicate that these parameters are informative when following populations longitudinally. Patients in Venezuela harbour more complex infections nowadays, and that criteria may allow separating a potentially imported case from a Venezuelan migrant that acquired malaria locally since MOI tend to be lower in other regions of Latin America.

## Supplementary information

**Additional file 1: Table S1. (A)** Complete list of *P. falciparum Pfk13* gene sequences available in databases. The codons associated with artemisinin resistance located at the propeller domain are shown. Venezuelan complete sequences (N=65) are labelled in purple. In this study, only the complete sequences were used for the analyses. **(B)** The mutation found in the Venezuelan samples and the other countries where these mutations were also found.

**Additional file 2: Table S2. (A)***Plasmodium falciparum* data and **(B)***P. vivax*: One-sided bootstrap test for differences in MOI. The test statistic (difference in estimated λ), and p-value of bootstrap test based on B=10,000 bootstrap repeats using bias correction and acceleration are shown. The lower and upper bounds of the 95% bias-corrected and accelerated (BCa) bootstrap condensed interval of the test statistic based on B=10,000 bootstrap repeats are also shown. All markers showed an increase in MOI.

**Additional file 3: Table S3. (A)***Plasmodium falciparum* and **(B)***P. vivax* data: Two-sided bootstrap test for differences in heterozygosity per marker. The test statistic (difference in heterozygosity), and p-value of bootstrap test based on B=10,000 bootstrap repeats using bias correction and acceleration are shown. The lower and upper bounds of the 95% bias-corrected and accelerated (BCa) bootstrap condensed interval of the test statistic based on B= 10,000 bootstrap repeats, as well as the p-value for a permutation test to compare the underlying frequency distributions using the differences in heterozygosity as test statistic, are also shown. The permutation test was approximated using 10,000 permutations. Note, that the permutation test will have relatively low power because of the employed test statistic. Namely, different frequency distributions can have similar heterozygosity values. Only some markers show a difference in MOI.

**Additional file 4: Figure S1.***Plasmodium falciparum* data: Frequency distribution of alleles per locus and year sampled (2003/2004 and 2018).

**Additional file 5: Figure S1. (B)***P. vivax data*: Frequency distribution of alleles per locus and year sampled (2003/2004 and 2018).

**Additional file 6: Table S4. (A)***Plasmodium falciparum* and **(B)***P. vivax* data: cumulated explained variance by PCAs. In *P. falciparum*, about 64% of the variance is explained by the first 4 components and in *P. vivax*, about 35% of the variance is explained by the first 4 components.

## Data Availability

Sequences were deposited in the GenBank with the accession numbers MT120216 to MT120303.

## Abbreviations

ALD: Asymmetric linkage disequilibrium
CC: Clonal complex
*Pfdhfr*: Bifunctional dihydrofolate reductase-thymidylate synthase
*Pfdhps*: Dihydropteroate synthase
dN: Nonsynonymous substitutions per nonsynonymous site
dS: Synonymous substitutions per synonymous site
G: Number of unique genotypes
He: Nei’s index of genetic diversity
IC: Informed consent
LD: Linkage disequilibrium
ML: Maximum-likelihood
MOI: Multiplicity of infection
MST: Minimum Spanning Tree-like
nLV: n-locus-variants-level
PCA: Principal component analyses
PCR: Polymerase chain reaction
*Pfcrt*: Chloroquine resistance transporter
PG: Number of private genotypes
S: Number of segregating sites
SD: Standard deviations
SE: Standard error
SMG: Sampled multilocus genotypes
SP: Sulfadoxine-pyrimethamine
STs: Sequence types
STRs: Standard microsatellite loci
TBS: Thick blood smears
