# Supplementary material for: Malaria in Venezuela: changes in the complexity of infection reflects the increment in transmission intensity
Source: Malar J. 2020 May 7;19:176. doi: 10.1186/s12936-020-03247-z (PMC7206825; doi:10.1186/s12936-020-03247-z)
Supplement: Supplementary file 2 — Additional file 2: Table S2. (A)Plasmodium falciparum data and (B)P. vivax: One-sided bootstrap test for differences in MOI. The test statistic (difference in estimated λ), and p-value of bootstrap test based on B=10,000 bootstrap repeats using bias correction and acceleration are shown. The lower and upper bounds of the 95% bias-corrected and accelerated (BCa) bootstrap condensed interval of the test statistic based on B=10,000 bootstrap repeats are also shown. All markers showed an increase in MOI. [file 12936_2020_3247_MOESM2_ESM.pdf]

Additional file 2: Table S2. (A) *Plasmodium falciparum* data and **(B)** *P. vivax*: One-sided bootstrap test for differences in MOI. The test statistic (difference in estimated λ), and p-value of bootstrap test based on B=10,000 bootstrap repeats using bias correction and acceleration are shown. The lower and upper bounds of the 95% bias-corrected and accelerated (BCa) bootstrap condensed interval of the test statistic based on B=10,000 bootstrap repeats are also shown. All markers showed an increase in MOI.

(A)

	test stat	p-val	low	up
Polyα	0.1786	0.0000	0.0604	0.5647
TAA81	0.2414	0.0000	0.1394	0.7641
TAA42	1.1177	0.0000	0.0000	2.5030
TAA109	0.4676	0.0000	0.1666	1.7883
ARA2	0.8051	0.0000	0.5374	1.4233
Pfg377	0.1631	0.0000	0.0616	0.0000
PfPK2	0.3213	0.0000	0.1772	0.9261
TAA60	0.4334	0.0000	0.2412	1.3629

(B)

	test stat	p-val	low	up
MS2	1.3124	0.0000	1.0414	1.9730
MS5	1.3842	0.0000	1.0945	2.0253
MS6	1.3914	0.0000	1.0730	1.8571
MS15	1.2938	0.0000	1.0076	1.7916
14.185	0.9236	0.0000	0.6839	1.3923
2.21	1.5549	0.0000	1.2356	1.9796
MS8	1.5297	0.0000	1.1935	2.0754
MS1	1.0688	0.0000	0.8358	1.6602
MS10	2.0073	0.0000	1.4941	2.5542
