# Supplementary material for: Malaria in Venezuela: changes in the complexity of infection reflects the increment in transmission intensity
Source: Malar J. 2020 May 7;19:176. doi: 10.1186/s12936-020-03247-z (PMC7206825; doi:10.1186/s12936-020-03247-z)
Supplement: Supplementary file 3 — Additional file 3: Table S3. (A)Plasmodium falciparum and (B)P. vivax data: Two-sided bootstrap test for differences in heterozygosity per marker. The test statistic (difference in heterozygosity), and p-value of bootstrap test based on B=10,000 bootstrap repeats using bias correction and acceleration are shown. The lower and upper bounds of the 95% bias-corrected and accelerated (BCa) bootstrap condensed interval of the test statistic based on B= 10,000 bootstrap repeats, as well as the p-value for a permutation test to compare the underlying frequency distributions using the differences in heterozygosity as test statistic, are also shown. The permutation test was approximated using 10,000 permutations. Note, that the permutation test will have relatively low power because of the employed test statistic. Namely, different frequency distributions can have similar heterozygosity values. Only some markers show a difference in MOI. [file 12936_2020_3247_MOESM3_ESM.pdf]

Additional file 3: Table S3. (A) *Plasmodium falciparum* and **(B)** *P. vivax data*: Two-sided bootstrap test for differences in heterozygosity per marker. The test statistic (difference in heterozygosity), and p-value of bootstrap test based on B=10,000 bootstrap repeats using bias correction and acceleration are shown. The lower and upper bounds of the 95% bias-corrected and accelerated (BCa) bootstrap condensed interval of the test statistic based on B= 10,000 bootstrap repeats, as well as the p-value for a permutation test to compare the underlying frequency distributions using the differences in heterozygosity as test statistic, are also shown. The permutation test was approximated using 10,000 permutations. Note, that the permutation test will have relatively low power because of the employed test statistic. Namely, different frequency distributions can have similar heterozygosity values. Only some markers show a difference in MOI.

(A)

	test stat	p-val	low	up	p-val perm
Polyα	-0.2284	0.0000	-0.3376	-0.0818	0.0000
TAA81	-0.4057	0.0250	-0.4828	-0.2388	0.0000
TAA42	0.5332	0.0000	0.4783	0.6011	0.0000
TAA109	0.3625	0.0000	0.2662	0.5663	0.0000
ARA2	-0.3928	0.0995	-0.4450	-0.2185	0.0000
Pfg377	-0.1709	0.0192	-0.2644	-0.0412	0.0000
PfPK2	0.1257	0.0001	0.0606	0.2163	0.0012
TAA60	0.0115	0.8476	-0.0844	0.1440	0.7814

(B)

	test stat	p-val	low	up	p-val perm
MS2	-0.0412	0.1437	-0.1190	0.0134	0.0000
MS5	-0.2637	0.0000	-0.3580	-0.1769	0.0000
MS6	0.0270	0.3944	-0.0407	0.0876	0.0361
MS15	0.0002	0.9830	-0.0773	0.0589	0.9916
14.185	-0.1097	0.0600	-0.2231	0.0034	0.0128
2.21	0.0543	0.0973	-0.0090	0.1440	0.0030
MS8	-0.0595	0.0317	-0.1118	-0.0043	0.0000
MS1	-0.1251	0.0008	-0.1977	-0.0557	0.0000
MS10	0.0511	0.2437	-0.0315	0.1724	0.0878
