# Supplementary material for: Malaria in Venezuela: changes in the complexity of infection reflects the increment in transmission intensity
Source: Malar J. 2020 May 7;19:176. doi: 10.1186/s12936-020-03247-z (PMC7206825; doi:10.1186/s12936-020-03247-z)

**Additional file 4: Figure S1A.** *Plasmodium falciparum* data: Frequency distribution of alleles per locus and year sampled (2003/2004 and 2018).

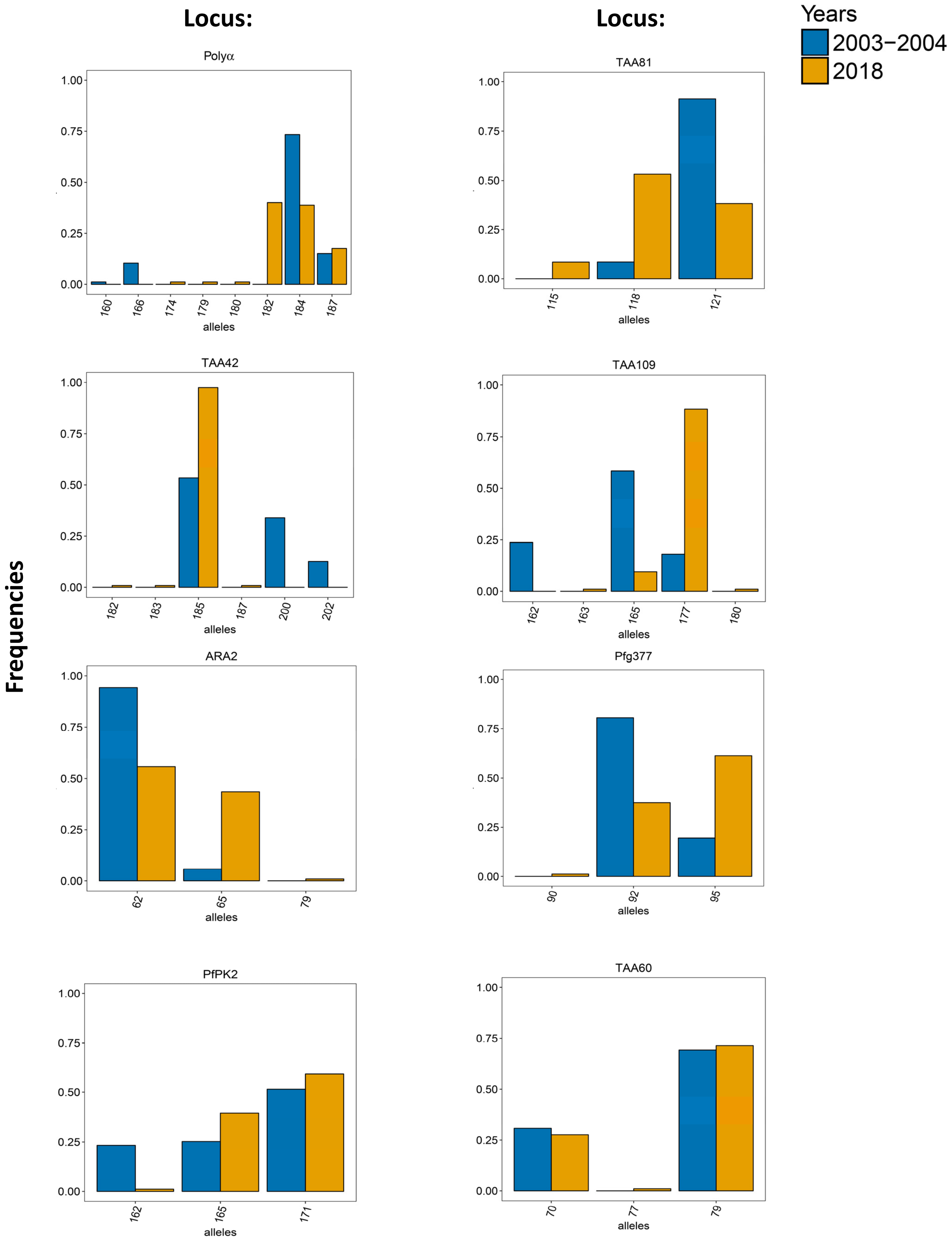

Supplement: Supplementary file 4 — Additional file 4: Figure S1.Plasmodium falciparum data: Frequency distribution of alleles per locus and year sampled (2003/2004 and 2018). [file 12936_2020_3247_MOESM4_ESM.pdf]
