# Supplementary material for: Malaria in Venezuela: changes in the complexity of infection reflects the increment in transmission intensity
Source: Malar J. 2020 May 7;19:176. doi: 10.1186/s12936-020-03247-z (PMC7206825; doi:10.1186/s12936-020-03247-z)

Additional file 5: Figure S1B. *Plasmodium vivax* data: Frequency distribution of alleles per locus and year sampled (2003/2004 and 2018).

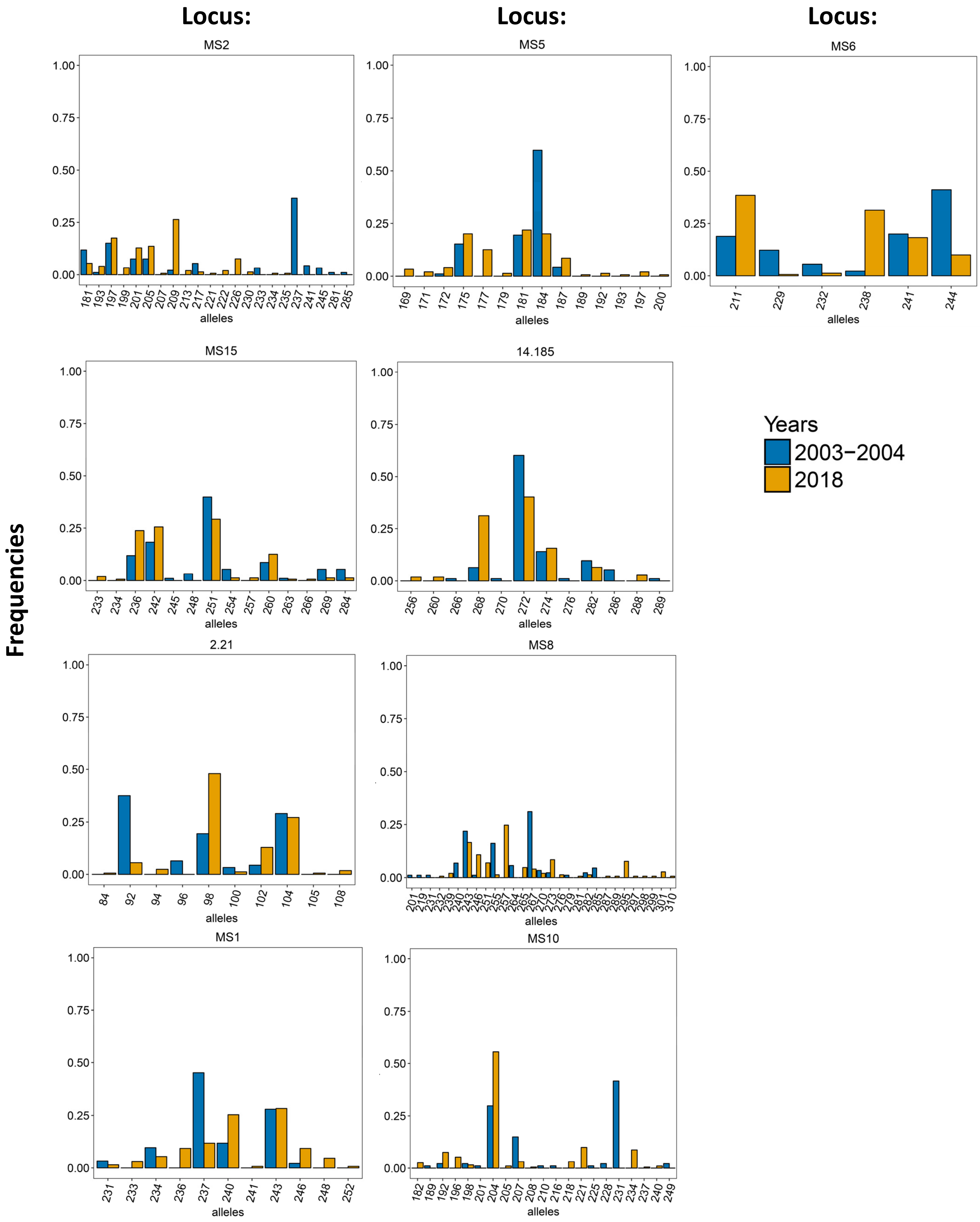

Supplement: Supplementary file 5 — Additional file 5: Figure S1. (B)P. vivax data: Frequency distribution of alleles per locus and year sampled (2003/2004 and 2018). [file 12936_2020_3247_MOESM5_ESM.pdf]
