# Supplementary material for: Malaria in Venezuela: changes in the complexity of infection reflects the increment in transmission intensity
Source: Malar J. 2020 May 7;19:176. doi: 10.1186/s12936-020-03247-z (PMC7206825; doi:10.1186/s12936-020-03247-z)
Supplement: Supplementary file 6 — Additional file 6: Table S4. (A)Plasmodium falciparum and (B)P. vivax data: cumulated explained variance by PCAs. In P. falciparum, about 64% of the variance is explained by the first 4 components and in P. vivax, about 35% of the variance is explained by the first 4 components. [file 12936_2020_3247_MOESM6_ESM.pdf]

Additional file 6: Table S4. (A) *Plasmodium falciparum* and **(B)** *P. vivax* data: cumulated explained variance by PCAs. In Pf, about 64% of the variance is explained by the first 4 components and in Pv, about 35% of the variance is explained by the first 4 components.

(A)

PCA	cum variance in %
1	0.2570
2	0.4228
3	0.5520
4	0.6421

(B)

PCA	cum variance in %
1	0.1645
2	0.2377
3	0.3008
4	0.3454
